# DNA Methylation: A Potential Biomarker of Chronic Obstructive Pulmonary Disease

**DOI:** 10.3389/fcell.2020.00585

**Published:** 2020-07-07

**Authors:** Lin-Xi He, Zhao-Hui Tang, Qing-Song Huang, Wei-Hong Li

**Affiliations:** ^1^School of Basic Medicine Sciences, Chengdu University of Traditional Chinese Medicine, Chengdu, China; ^2^Department of Respiratory, Affiliated Hospital of Chengdu University of Traditional Chinese Medicine, Chengdu, China

**Keywords:** DNA methylation, COPD, biomarker, tissue specificity, lung cancer

## Abstract

Chronic obstructive pulmonary disease (COPD) is a serious public health concern worldwide. By 2040, 4.41 million people are estimated to expire annually due to COPD. However, till date, it has remained difficult to alter the activity or progress of the disease through treatment. In order to address this issue, the best way would be to find biomarkers and new therapeutic targets for COPD. DNA methylation (DNAm) may be a potential biomarker for disease prevention, diagnosis, and prognosis, and its reversibility further makes it a potential drug design target in COPD. In this review, we aimed to explore the role of DNAm as biomarkers and disease mediators in different tissue samples from patients with COPD.

## Introduction

Chronic obstructive pulmonary disease (COPD) is a chronic progressive disease (Rabe and Watz, 2017). Its pathological features mainly include irreversible airway obstruction, mucus secretion, and inflammation (Celli and MacNee, 2004). Fifty years ago, it had prompted the establishment of the Department of Pulmonary Diseases. Unfortunately, with the world’s population aging rapidly, COPD has become an increasingly serious problem (Crapo, 2019). The Global Burden of Disease Study (GBDS) had estimated ∼299 million patients to have COPD, worldwide, in 2017 (GBD 2017 Disease and Injury Incidence and Prevalence Collaborators, 2018). In 2016, ∼2.93 million patients across the world died of the disease due to its high incidence rate. By 2040, the number of deaths due to COPD is expected to be double of that in 2016, which implies ∼4.41 million deaths due to COPD (Foreman et al., 2018). The above data, however, does not include the patients with COPD who died of cardiovascular disease (Calverley et al., 2007), and those with some cardiovascular diseases along with airflow restriction (Franssen et al., 2016). Therefore, the global mortality caused by COPD is remarkably underestimated, and in near future, it could pose a serious public health concern. For many years, the clinical treatment of COPD has focused on relieving patients’ symptoms, and improving their health and quality of life. However, till date, it has remained very difficult to alter the progress of this disease through treatment. COPD is a heterogeneous disease, which may need precise and personalized therapeutic approach (Heaney and McGarvey, 2017). Moreover, early diagnosis of the disease would be necessary, before the occurrence of disability or irreversible lung structural changes (Lowe et al., 2019). The best way to solve this problem would be to explore the biomarkers of COPD and identify new therapeutic targets.

COPD is a complex disease affected by environmental factors (smoking or drugs); therefore, its pathological mechanism is driven by genetic and epigenetic variation (Qiu et al., 2012; Vucic et al., 2014). Through genome-wide genetic association studies (GWAS), many COPD-susceptible gene loci, such as *FAM13A* (Cho et al., 2010; Chen et al., 2015), *CHRNA* 3/5 (Pillai et al., 2009; Hardin et al., 2012) and *HHIP* (Pillai et al., 2009), had been identified previously (Young et al., 2008, 2010, 2011; Hancock et al., 2010; Lambrechts et al., 2010; Repapi et al., 2010; Van Durme et al., 2010; Chappell et al., 2011). Interestingly, many single nucleotide polymorphisms related to COPD were found in the non-coding region and intron regulatory regions of the gene; this result was consistent with many complex diseases (Hindorff et al., 2009; Maurano et al., 2012). These single nucleotide polymorphisms may be speculated to be involved in the regulation of epigenetic modification. Some differential methylated sites (CPGs), unrelated to GWAS but closely related to those regarding smoking (the main exposure factor of COPD), have potential significance in COPD susceptibility (Qiu et al., 2012, 2015; Vucic et al., 2014). Therefore, genetic variation and environmental exposure mediated by epigenetic modification may enhance the risk of COPD (Qiu et al., 2012).

Epigenetic modification mainly includes DNA methylation and various post-translational modifications of histones (histone PTM, including histone acetylation, methylation, phosphorylation, ubiquitination and sulfonation). Among them, COPD is mainly related to histone acetylation. Histone deacetylase is the key enzyme to control the proinflammatory cytokines related to COPD (Bowman et al., 2009). The balance of histone acetylation/deacetylation in COPD patients will shifted toward acetylation (Szulakowski et al., 2006). The imbalance of histone acetylation and deacetylation changes the nucleosome structure in the transcription of inflammatory cytokines, affects the expression of inflammatory genes (Mroz et al., 2007, 2008). This leads to the change of gene expression profile in COPD patients (Szulakowski et al., 2006).

DNA methylation (DNAm) is a tissue-specific epigenetic modification involved in the regulation of gene expression (Vucic et al., 2014). It plays an important role in many chronic complex diseases, like cancer (Luo et al., 2020) and aging (Horvath and Raj, 2018), and is the basis of normal physiological development. Many studies, till date, have confirmed DNA methylation, related to COPD, to be a potential biomarker for disease prevention, diagnosis, and prognostic evaluation (Qiu et al., 2012; Sundar et al., 2017). Furthermore, its reversibility makes it an attractive candidate for designing disease biomarkers and drugs. In this review, we focused on the role of DNAm as biomarkers and disease mediators in different tissue samples from patients with COPD.

## Biomarker in Blood

**FIGURE 1:**
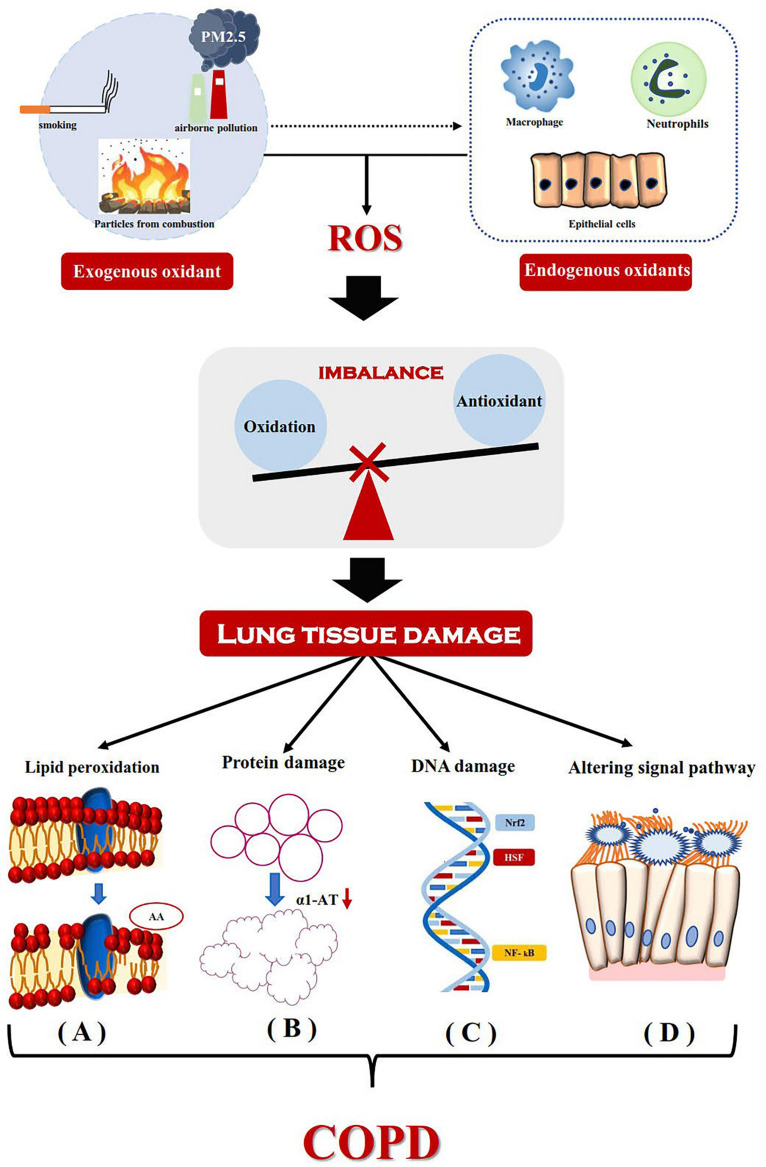
Mechanism for the development of COPD driven by oxidative stress. Exogenous oxidants from the environment and endogenous oxidants (released by activated neutrophils, alveolar macrophages and epithelial cells) lead to the production of reactive oxygen species and cause lung tissue damage. It mainly includes lipid peroxidation, Protein damage, DNA damage and Altering signal pathway. **(A)** Lipid peroxidation results in the hydrolysis of phospholipids in the cell membrane and changes in the structure and permeability of the cell membrane. Arachidonic acid produced by hydrolysis of phosphoric acid molecules, its metabolites thromboxane, prostaglandin E and leukotriene C4 are involved in the inflammatory process. **(B)** The inactivation of α1-AT breaks the balance between protease and anti-protease, which do great damage to elastin and eventually leads to emphysema. **(C)** Gene expression of inflammatory mediators induced by DNA damage. **(D)** Oxidative stress changes TNF-α-mediated signaling pathway, which leads to increased mucus secretion and ciliated cell dysfunction. ROS, reactive oxygen species; AA, arachidonic acid; α1-AT, alpha-1-antitrypsin; Nrf2, nuclear factor erythroid-2-related factor2; HSF, heat shock transcription factor; NF- kB, Nuclear Factor-κB.

Blood samples from patients with COPD had previously been reported to show hypomethylation of DNA, which is related to severity of the disease (Zinellu et al., 2017c). Some studies had suggested DNA hypomethylation to be related to oxidative stress (OS) via various mechanisms (Fomenko et al., 2007; Hitchler and Domann, 2007; Franco et al., 2008). The redox state of cells, with higher degree of oxidation, may lead to the reduction of DNAm through redox regulation of related enzymes, such as SAM-dependent methyltransferase (Fomenko et al., 2007). In an environment with higher level of oxidation, the activity of methionine adenosyltransferase is decreased, which in turn catalyzes the addition of methionine to adenosine to synthesize SAM (Pajares et al., 1992). The important role of OS in COPD has now been confirmed (Figure 1; Zinellu et al., 2016a, b, 2016); the characteristics of hypoxia in COPD can enhance OS by increasing ROS production at the level of mitochondrial respiratory chain (Chandel and Schumacker, 2000; Hoppeler et al., 2003). In contrast, Angelo Zinellu et al. had reported DNA hypomethylation in the blood of patients with COPD to be related to disease severity, although there was no significant correlation between DNAm and OS index (Zinellu et al., 2017c). This may be due to the relatively small sample size of the study or the lack of severely unstable patients with COPD (particularly with high oxidative stress). At the same time, Qiu et al. (2012) had screened 27578 CpG loci in subjects with COPD, by using the HumanMethylation27 array (Illumina, San Diego, CA, United States). A total of 349 CpG loci was found to be significantly related to the susceptibility and severity of COPD. Gene ontology analysis, based on these CpGs, suggested the involvement of immune and inflammatory system pathways, stress response, and external stimulation. Hypomethylation of *SERPINA1* and fucosyltransferase-7 (*FUT7*) genes was found to be closely related to COPD and pulmonary dysfunction. Recently, the haplotypes of two major alleles of *SERPINA1* were found to be related to reduced risk of COPD (Ponce-Gallegos et al., 2019). *SERPINA1* gene encodes a sialic acid protein called α-1 antitrypsin (AAT), whose main function is to protect the lower respiratory tract from degradation by neutrophil elastase (Salahuddin, 2010). AAT deficiency is generally believed to be the most important genetic risk factor of COPD. Decrease of α-1 antitrypsin level in circulation leads to the increase of elastase activity in pulmonary neutrophils; it is the imbalance of protease–antiprotease that leads to lung remodeling (Brode et al., 2012). *FUT7* encodes sialyl Lewis X (SLeX), which can promote leukocyte migration to inflammatory tissue (Miyashiro et al., 2004). Furthermore, the SLeX is a ligand of E-selectin (ES), which is increased in patients with COPD (Di Stefano et al., 1994). Therefore, future research should focus on whether the relative hypomethylation of *FUT7* could change the peripheral expression of SLex and eventually lead to the transfer of neutrophils to the lung. If this hypothesis could be confirmed, it will be another exciting and biologically credible way. Some new differential methylation sites related to COPD had been identified in the DNA of Korean blood samples, including cg03559389 (*DIP2C*) and a rare variant (rs140198372) of cg19904425 (*SERPINA 12*) (Lee et al., 2017). Among them, the *DIP2C* mutation was found in lung cancer samples (Gao et al., 2013) and *SERPINA12* was reported to be related to airflow restriction (Jackson et al., 2016). For the systematic evaluation of COPD as well as DNAm of peripheral blood in a population-based study, no consistent CpG locus was found in the study of COPD status or that of lung function value (Machin et al., 2017). Large-scale research on vertical design to solve reverse causality may prove to be a more fruitful research approach.

Blood biomarkers are useful for the assessment of systemic properties of COPD (Faner et al., 2014). Compared to lung tissue samples, the risk in blood sampling is lower. In addition, sampling of blood samples can be easily repeated, which is beneficial for longitudinal monitoring of disease progression. Compared to other minimally invasive biological samples (such as urine or sputum), blood samples have a wider range of application, more repeatability, and are less time-consuming. These characteristics make blood sampling more practical in large-scale research (Regan et al., 2019).

## Biomarker in Sputum

Methylation of *P16* and *GATA4* promoter in sputum samples was assumed to be an early biomarker of COPD (Sood et al., 2010; Meek et al., 2015). *GATA4* is a transcription regulator for many cell cycle genes (Rojas et al., 2008). In humans, it is a crucial transcription factor for normal lung development (Ackerman et al., 2007). Moreover, methylation of *GATA4* gene has been found to regulate oxidative stress and/or airway inflammation, which is related to the health status of patients with COPD (Yao and Rahman, 2011). Therefore, methylation of *GATA4* gene in sputum may independently predict the health status of patients with COPD (Meek et al., 2015). P16 mediated cell cycle arrest and senescence (Takahashi et al., 2006), The absence of p16 activated the accumulation of Akt and cyclin D, upregulated IGF1 pathway, promoted the proliferation and regeneration of aecii, and finally effectively prevented the cigarette induced emphysema (Cottage et al., 2019). Of course, there are also some researchers who are opposed to the protective effect of p16, but the sample size of relevant studies is very small (only 3 cases), and the reliability of the results needs further study (Sundar et al., 2018).

The methylation pattern of tumor suppressor gene (TSG) promoter was studied in sputum samples of patients with COPD; they included cyclin dependent kinase inhibitor 2A (Berger and Bardeesy, 2007), *MGMT* (Belinsky et al., 2002), and *CDH1* (Elloul et al., 2006). The extent of methylation in *CDKN2A* and *MGMT* was significantly higher. Induction of abnormal methylation in TSG in sputum samples could be a useful tool for early diagnosis of COPD (Guzmán et al., 2012).

## Biomarker in Tracheal Epithelium and Lung Tissue

COPD is a heterogeneous inflammatory disease of the airways, alveolus, and microvasculature (Rabe and Watz, 2017). Since DNAm in lung tissue (Cheng et al., 2016; Morrow et al., 2016) and small airway tissue (Vucic et al., 2014) is closely related to COPD, tissue-specific DNAm may be considered to occur in COPD. Unlike the hypomethylation pattern of DNAm in blood (Qiu et al., 2012), most differentially methylated genes in COPD-associated small airway epithelia (SAE) are hypermethylated. Although collection of samples of airway and lung tissues is difficult, a large volume of literature exists regarding DNAm markers in tracheal epithelium and lung tissues (Table 1).

**TABLE 1:** Potential DNA methylation markers in tracheal epithelium and lung tissue of COPD.

References	Gene	Tissue
Clifford et al. (2018)	OAT, GRIK2	Parenchymal fibroblasts
Morrow et al. (2018)	KCNK3, EEFSEC, PIK3CD, DCDC2C, TCERG1L, FRMD4B, IL27	Lung tissue
Qiu et al. (2018)	Klotho	Human bronchial epithelial
Sundar et al. (2017)	NOS1AP, TNFAIP2, BID, GABRB1, ATXN7, THOC7	Lung tissue
Barnawi et al., 2017	S1PR5	Alveolar macrophages
Song et al. (2017)	SPDEF, FOXA2	Bronchial tissue (primary airway epithelial cells)
Morrow et al. (2016)	FRMD4A, THSD4, C10orf11	Lung tissue
Yoo et al. (2015)	EPAS1	Lung tissue

In view of the common genetic and epigenetic variation of COPD, some researchers follow the results of GWAS as a guide to prioritize the identified loci related to COPD (Morrow et al., 2018) with epigenetic annotation (Kundaje et al., 2015), and highlight the mechanisms related to complex traits. The genetic control of DNAm has been observed in a variety of complex traits and diseases (Olsson et al., 2014; Hannon et al., 2016; Bonder et al., 2017). Morrow et al. (2016) had analyzed the whole genome DNAm of homogenous lung tissue samples from patients with COPD. The differential methylation sites were integrated with previous genome-wide studies and four of them, namely *CHRM1*, *DTX1*, *GLT1D1*, and *C10orf11*, were focused upon in the study (Morrow et al., 2016). The *CHRM1* is known to be associated with airway constriction, and a small candidate gene study had shown it to be associated with asthma (Maeda et al., 2006) and nicotine dependence (Lou et al., 2006). In addition, the role of other muscarinic receptor genes had also been previously reported in COPD (Cherubini et al., 2016).

In the lung parenchyma of patients with COPD, researchers had found differentially methylated genes to be closely related to the top typical pathways (such as β-γ signal or cancer mechanism), diseases and disorders (tissue damage and abnormality, cancer and respiratory system diseases), and molecular and cellular functions (Sundar et al., 2017). Genomic DNAm analysis had confirmed the changes in DNAm status of suggestive genes such as *NOS1AP*, *TNFAIP2*, *BID*, *GABRB1*, *ATXN7*, and *THOC7* in COPD-lung tissue, and further verified the changes by pyrosequencing. DNAm was suggested to possibly play a key role in the regulation of gene expression related to molecular pathways and cellular processes in COPD (Yao and Rahman, 2011). Small airways are the main sites of airflow obstruction in patients with COPD; therefore, detection of markers in small-airway epithelium of patients with COPD could have important biological and clinical significance. Three pathways in SAE were found to have potential significance in the pathogenesis of COPD, namely phosphatase and tensin homolog (PTEN) signal, NF-E2-related factor 2 (Nrf2) mediated oxidative stress, and interleukin-17F(IL-17F) inflammatory response pathway (Vucic et al., 2014).

DNAm has a high level of cell-type specificity, there are some studies on DNAm of individual cell types in patients with COPD. Clifford et al. (2018) had studied the genomic DNAm of fibroblasts isolated from lung parenchyma and airway, and found DNAm to be related to differential gene expression only in the parenchymal fibroblasts. In addition, 359 individual differential CpG loci were found in the parenchymal fibroblasts. The methylation sites of OAT and GRIK2 genes significantly increased their expression in COPD cells (Clifford et al., 2018). Barnawi et al. (2017) had found phagocytosis of apoptotic cells by alveolar macrophages in patients with COPD to be controlled by epigenetic regulation, and decrease of methylation to possibly be the cause of increased S1PR5 gene expression in alveolar macrophages and of the defect in COPD-related efferent cells. Notch-mediated hypermethylation of Klotho in alveolar macrophages and airway epithelial cells inhibited the expression of Klotho and promoted inflammatory response and apoptosis in COPD (Qiu et al., 2018). Demethylation of NF-κ B-mediated pathway gene is related to the deterioration of COPD. However, TET1/2 plays an important role in the regulation of DNAm and production of cytokine/chemokine by *NF-*κ *B*, *STAT3*, *IKK*, and *NIk* genes in A549 cells (Kaur and Batra, 2020).

Goblet cell metaplasia is a common feature of COPD, which is related to mucus hypersecretion. SAM-pointed domain, a transcription factors containing ETS like factor (SPDEF) and forkhead box protein A2 (FOXA2), regulates the differentiation of goblet cells. Airway mucin 5AC, secreted by SPDEF, is aggravated the differentiation of goblet cells and mucin production (Park et al., 2007; Chen et al., 2009; Rajavelu et al., 2015), while FOXA2 effectively inhibited the differentiation of pulmonary goblet cells (Tang et al., 2013). Song et al. (2017) had found hypermethylation of CPG-8 in the promoter of *SPDEF* and hypomethylation of CpG-14 and CpG-15 in *FOXA2*, was identified the abnormal methylation of *SPDEF* and *FOXA2* during the differentiation of goblet cells is the basic factor of mucus hypersecretion in COPD, thus providing a new approach to understand mucus hypersecretion from the perspective of epigenetics.

Some researchers had identified key regulatory factors in lung samples of patients with COPD by integrating functional genome, epigenetic data, and higher-order phenotype data (Yoo et al., 2015). Endothelial PAS domain protein 1 (*EPAS1*) was found to be the only key regulatory factor with a significant overlap of multiple gene sets related to COPD, and the protein level of *EPAS1* was low in the lung tissue of patients with COPD (Yoo et al., 2015). *EPAS1* is a hypoxia responsive transcription factor, also known as hypoxia inducible factor 2 α (HIF2A) (Ema et al., 1997; Tian et al., 1997). Its expression in lung and endothelial cells was higher than that of hypoxia inducible factor 1 α (HIF1A). The decrease of *EPAS1* expression observed in COPD actually lead to inadaptability of hypoxia response (Kent et al., 2011). Therefore, it would be highly significant to understand the role and mechanism of *EPAS1* in the treatment of diseases.

The above study found significant levels of abnormal DNAm to be different across lung tissues and cell types. It depends on the number of samples (Sundar et al., 2017), the integration method of gene expression (Yoo et al., 2015), and the integration scheme with GWAS results (Morrow et al., 2016).

## Discussion

DNAm is obviously a potential biomarker for disease prevention, diagnosis, and prognosis. Owing to its reversibility, it has been widely considered as a biomarker and drug design target in COPD. While it has huge prospects in research, there are some limitations in the studies as well. First, the studies were limited by the type of sample: either they were conducted in blood, related to blood biomarkers of the disease (although the transformation of lung pathology was limited), or they are carried out in the lung tissue, where the cell type-specific methylation spectrum could hide the disease-related changes. Stueve et al. (2017) confirmed that DNA methylation from peripheral blood can serve as a surrogate marker for DNA methylation in lung tissue, which shows that it is feasible to search for cross tissue DNA methylation markers (especially in blood and lung tissue) in COPD patients. Moreover, mixed cell population in the whole tissue might complicate the study of the mechanism of cell type-specificity in subsequent diseases. Second, cross-sectional studies hindered the causal relationship between methylation changes and COPD status or lung function level. We are not sure whether these are prior to COPD or the result of COPD. Finally, the studies focused on the European population, with few methylation and transcriptome data from other populations, such as the Asians.

Nevertheless, DNAm as a potential biomarker of COPD, has a broad research prospect. First of all, COPD, as a non-neoplastic lesion, often occurs simultaneously with lung cancer (Tockman et al., 1987; Mannino et al., 2003). Hypermethylation of *IL-12RBETA2* and *WIF-1* has been found frequently in the transition from COPD to lung cancer (Suzuki et al., 2010). Therefore, COPD and lung cancer are suggested to be an epigenetic continuum characterized by the accumulation of methylation markers over time. In addition, large-scale methylation studies may help to reveal the details of epigenetic development of COPD into lung cancer, which would be of great significance to public health. Second, the discovery of CRISPR-Cas9 has promoted the development of epigenetic editing (Wu et al., 2018). Its advantages in targeting, safety, and preventing non-targeting effect make epigenetic editing, based on epigenetic markers, feasible. In addition, unlike genome editing, epigenetic editing does not lead to permanent changes in the genome. The core of its function is to correct abnormal epigenetic markers to affect the expression or function of specific genes. This could provide a very effective method for the treatment of COPD in future. It is also urgent to obtain dynamic and persistent information of epigenetic markers for different cell types.

## Author Contributions

L-XH, Q-SH, and W-HL designed the manuscript. L-XH wrote the first draft of the manuscript. Z-HT, Q-SH, W-HL, and L-XH wrote specific sections of the manuscript. All authors contributed to the article and approved the submitted version.

## Conflict of Interest

The authors declare that the research was conducted in the absence of any commercial or financial relationships that could be construed as a potential conflict of interest.
